# Blunted Expected Reward Value Signals in Binge Alcohol Drinkers

**DOI:** 10.1523/JNEUROSCI.2157-21.2022

**Published:** 2023-08-02

**Authors:** Serenella Tolomeo, Alex Baldacchino, J. Douglas Steele

**Affiliations:** ^1^Institute of High Performance Computing (IHPC), Agency for Science, Technology and Research (A*STAR), 1 Fusionopolis Way, #16-16 Connexis, Singapore 138632; ^2^Division of Population and Behavioral Science, Medical School, University of St Andrews, KY16 9TF St Andrews, Scotland; ^3^Division of Imaging Science and Technology, Medical School, University of Dundee, DD1 4HN Dundee, Scotland

**Keywords:** binge drinking, model-based fMRI, orbitofrontal, prediction error signal, reinforcement learning, value

## Abstract

Alcohol-related morbidities and mortality are highly prevalent, increasing the burden to societies and health systems with 3 million deaths globally each year in young adults directly attributable to alcohol. Cue-induced alcohol craving has been formulated as a type of aberrant associative learning, modeled using temporal difference theory with an expected reward value (ERV) linked to craving. Clinically, although harmful use of alcohol is associated with increased time spent obtaining and using alcohol, it is also associated with self-neglect. The latter implies that the motivational aspects of nonalcohol stimuli are blunted. Using an instrumental learning task with non-alcohol-related stimuli, here, we tested hypotheses that the encoding of cue signals (ERV) predicting reward delivery would be blunted in binge alcohol drinkers in both sexes. We also predicted that for the binge drinking group alone, ratings of problematic alcohol use would correlate with abnormal ERV signals consistent with between groups (i.e., binge drinkers vs controls) abnormalities. Our results support our hypotheses with the ERV (nonalcohol cue) signal blunted in binge drinkers and with the magnitude of the abnormality correlating with ratings of problematic alcohol use. This implies that consistent with hypotheses, the motivational aspects of non-alcohol-related stimuli are blunted in binge drinkers. A better understanding of the mechanisms of harmful alcohol use will, in time, facilitate the development of more effective interventions, which should aim to decrease the motivational value of alcohol and increase the motivational value of non-alcohol-related stimuli.

**SIGNIFICANCE STATEMENT** Allostasis theory predicts specific abnormalities in brain function and subjective experiences that occur when people develop drug problems including addiction. Cue-induced alcohol craving has been formulated as a type of aberrant associative learning, modeled using temporal difference theory with ERV linked to craving. Here, we used an instrumental learning task with non-alcohol-associated stimuli to test hypotheses that the encoding of nonalcohol cue signals (ERV) and reward prediction error signals showed blunting in binge alcohol drinkers. We conclude that fMRI can be used to noninvasively test allostasis and associative learning theory predictions in binge drinkers.

## Introduction

Binge alcohol drinking involves the consumption of large quantities of alcohol in a short period and is a pattern of consumption usually acquired in youths (World Health Organization, 2018). Individuals who regularly binge drink are exposed to immediate and long-term societal and medical consequences and are at substantially increased risk of developing alcohol dependency (Courtney and Polich, 2009).

**Figure 1.**
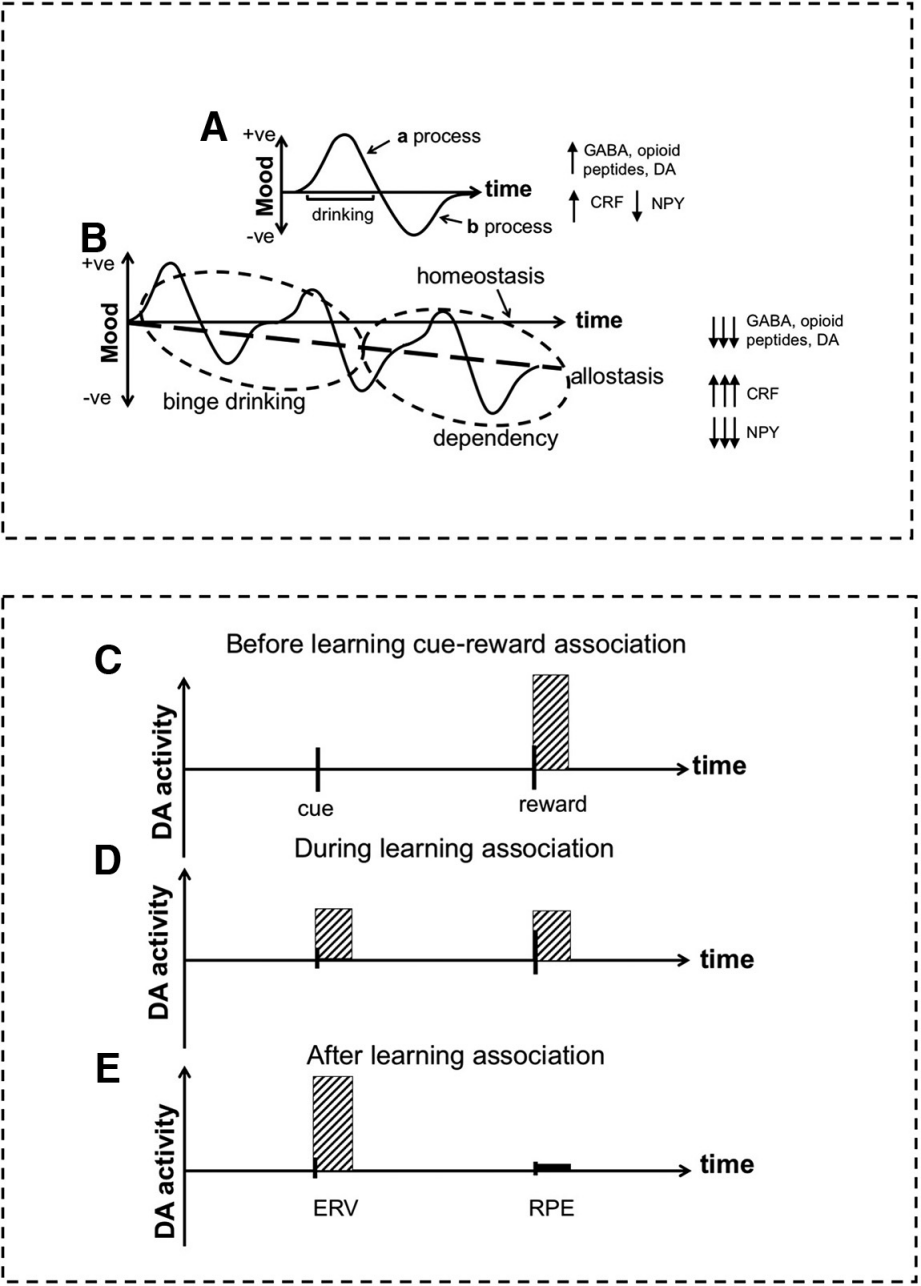
Allostasis theory and associative learning theory. ***A***, Single first-episode alcohol exposure with positive (+) mood (***a*** process) during drinking followed by a postintoxication hangover comprising negative (−) mood (***b*** process) With repeated episodes of binge drinking (intoxication), the ***a*** process diminishes, and the depth and duration of the ***b*** process increases with low mood and anxiety. ***B***, Frequent repeated alcohol use in which the ***b*** process does not have time to fully return to homeostasis results in mood drifting downward and hyperkatifeia, defined as a negative-valenced longer-duration mood state with stress vulnerability (alcohol dependency). Figure adapted from multiple sources (Koob and Le Moal, 2001; Koob, 2003; Koob and Schulkin, 2018; Tolomeo et al., 2021). Associative learning occurs as a series of trials comprising cue exposure (CS) followed by the delivery (or not) of a reward (US). ***C***, Before learning the CS-US association, dopamine firing occurs at the time of reward delivery and not at time of the cue. ***D***, As the CS-US association is learned, dopamine firing diminishes at the time of reward delivery and appears at the time of the cue predicting reward delivery. ***E***, When the association is learned, dopamine activity maximally occurs at the time of the cue and minimally at the time of reward delivery. Instrumental learning is a type of associative learning that involves an active choice between different cues with reward delivery contingent on the choice. According to the TD model of associative learning (Pessiglione et al., 2006), the dopamine signal at the time of the cue is the ERV, and the dopamine signal at the time of reward delivery is the RPE with the latter defined as RPE = *r* – ERV. Our previous work used *r* for fMRI analyses, and we reported blunting of this signal consistent with allostasis theory (Tolomeo et al., 2021). From TD theory this implies the RPE and consequently ERV signals should also be blunted, which we tested in the present study. Alcohol and drugs are a pharmacological type of reward, and consumption of these may cause pharmacologically enhanced *r* resulting in abnormally increased ERVs for alcohol/drug cues (Redish, 2004), enhancing their salience (McClure et al., 2003). CRF, Corticotrophin releasing factor; DA, dopamine; NPY, neuropeptide Y.

Progressive stages of harmful alcohol use, from occasional to frequent binge drinking to alcohol dependency, can be characterized by the allostasis theory (Koob and Le Moal, 2001), that is, progressive adaptation of the brain to repeated alcohol exposure, with downregulation of the reward system and upregulation of the stress-negative emotional system (Fig. 1). Problematic alcohol use begins with impulsive binge alcohol drinking driven primarily by short-term pleasurable effects, which causes adaptation of the brain over time and a shift from impulsive hedonic alcohol use to compulsive (Koob and Volkow, 2010; Tolomeo et al., 2018) avoidance of hypohedonia with increased stress vulnerability—hyperkatefia (Koob and Le Moal, 2001). Associated abnormalities in neurotransmitters, including dopamine, GABA, and glutamate, have been reported in preclinical (Koob and Schulkin, 2018) and clinical (Tolomeo et al., 2021) studies.

Allostasis theory emphasizes progressive blunting of brain reward responses. In contrast however, PET studies of drug cue exposure in alcohol and other drug dependencies have consistently reported increased dopamine release compared with healthy controls (Volkow et al., 2006; Wong et al., 2006; Cox et al., 2017), yet blunted dopamine release at the time of drug delivery compared with healthy controls (Volkow et al., 1997; Martinez et al., 2005, 2007). Increased dopamine release at the time of cue exposure has been linked to subjective craving or wanting the drug (Sell et al., 2000; Volkow et al., 2006; Saunders et al., 2013). As discussed later, experimental evidence from studies testing allostasis theory predictions and evidence from PET imaging studies on drug cue exposure may both be accommodated by associative learning theory (Fig. 1). This theory highlights the importance of (1) discriminating studies using alcohol delivery and alcohol-related cues (Claus et al., 2011) from studies using nonpharmacological natural rewards and non-alcohol-associated cues (Tolomeo et al., 2021) and (2) the importance of discriminating brain activity at the time of cue exposure from the time of reward delivery (Fig. 1).

Instrumental reward learning, a type of associative learning (Fig. 1), has been intensively studied over decades in healthy animals and humans with regard to both behavioral decision-making (Ferster and Skinner, 1957) and brain activity (Pessiglione et al., 2006). Invasive depth electrode recordings in awake behaving nonhuman primates revealed a pattern of dopamine activity in the ventral tegmental area during instrumental learning conforming to the predictions of temporal difference (TD) theory (Schultz et al., 1997). Later work reported the same signals measured noninvasively in healthy humans using model-based fMRI (Pessiglione et al., 2006). Similar learning models have been proposed for addiction (McClure et al., 2003; Zhang et al., 2009; Berridge, 2012).

Previously we reported a study on binge alcohol drinking that used an instrumental reward learning task with non-alcohol-related stimuli and fMRI to test allostasis theory–derived hypotheses (Tolomeo et al., 2021). Here, we instead used a TD model-based fMRI approach to analyze the same data, testing hypotheses that (1) cue signals for nonalcohol rewards [expected reward value (ERV) signals; Fig. 1] and reward prediction error (RPE) signals (Fig. 1) for delivery of non-alcohol-associated rewards are blunted in binge drinkers compared with controls and (3) abnormalities in these signals correlate with ratings of problematic alcohol use for the binge drinking group alone. Based on our previous work (Gradin et al., 2011) we predicted that abnormal ERV and RPE signals would be present in the amygdala-hippocampal complex and nucleus accumbens, respectively. GABA and glutamate can be measured noninvasively in humans using magnetic resonance spectroscopy and are implicated in reward value encoding (Jocham et al., 2012). We therefore predicted iii) that binge drinking would be associated with downregulation of GABA and/or upregulation of glutamate and correlate with ERV and RPE signal abnormalities in binge drinkers.

## Materials and Methods

### Participants

The East of Scotland Research Ethics Service (14/ES/0061) approved our study, and each participant provided written informed consent. We chose to study binge alcohol drinkers because of brain structure abnormalities associated with alcohol dependency (Squeglia et al., 2014) complicating the interpretation of results, and we considered binge drinking on a continuum with dependency (Fig. 1).

A sample size calculation was conducted before the start of the study using G*Power software (version 3.1.9.7). Considering an alfa level of 0.05, a total sample size of 57 was large enough to detect effect sizes (Cohen's *d* = 0.5) for a two-tailed *t* test including two groups (binge and controls). Fifty-seven subjects were recruited for a binge drinking group of 20 males and 18 females, all of whom described binge drinking every weekend. Half of this group were scanned before the weekend on a Friday, the others after the weekend on a Monday, with alternate assignments as recruitment progressed. This meant half the weekend binge drinkers were scanned on a Friday (with the longest time from last drinking) and half were scanned on a Monday (with the shortest time from last drinking) to test for increased fMRI and spectroscopic abnormalities in Monday binge drinkers. A group of 19 healthy controls (13 males, 6 females) were also scanned. Controls were assessed for past binge drinking or dependence and for any current or past psychiatric illness and neurologic disease. None of the subjects satisfied criteria for alcohol or other drug dependence and none were taking medications. All volunteers had normal or corrected-to-normal vision, and none had a history of neurologic problems. Data from one control subject was excluded because of movement during scanning. Data from the remaining 56 participants were therefore used in all subsequent analyses.

### Behavioral paradigm

**Figure 2.**
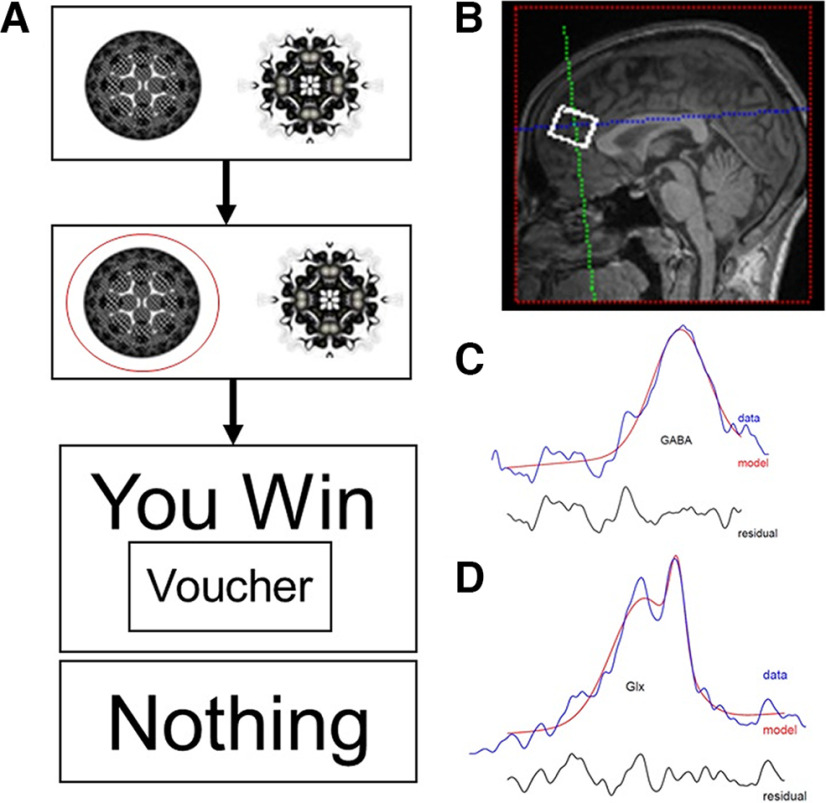
Behavioral paradigm and MR spectroscopy. ***A***, The reward–gain instrumental learning task. ***B–D***, Anterior mid-cingulate cortex region (***B***) selected for (***C***) GABA and (***D***) GLX measurement. GABA = Gamma-Amino-Butyric Acid; GLX = Glutamate-glutamine.

A task optimized for fMRI use with clinical groups was used (Gradin et al., 2014; Johnston et al., 2015; Tolomeo et al., 2021; Fig. 2). Each type of trial was associated with one of two pairs of fractal images (shaped as circles, squares, or triangles). The order of the associations with different picture pairs was randomized. At the beginning of each trial, a fractal pair was presented, and the participant had to select the left or right fractal picture by pressing the button. Once a fractal picture had been chosen, it appeared circled in red, and later the outcome was displayed. The paradigm has two relevant outcomes, reward delivery (a win message) and lack of reward delivery (a nothing message). Volunteers were told the aim of the task was to maximize winning by trial and error, and based on their performance (the accumulated points), they would receive a gift voucher. The probability of win/nothing fractal pairs had a fixed high reward probability (70%) and a fixed low reward probability (30%). Each session had 60 trials with each session lasting 13 min in total and three sessions per subject.

### Rating scales

The Alcohol Use Disorders Identification Test (AUDIT; Bohn et al., 1995) was used to help identify binge drinkers, diagnosed according to the definition of the National Institute on Alcohol Abuse and Alcoholism, which is consumption of alcohol to a blood alcohol level of 0.08 × g/dl, which typically occurs after four drinks for women and five drinks for men when consumed in 2 h. The Severity of Alcohol Dependence Questionnaire (SADQ) was also used to assess dependence symptoms (Stockwell et al., 1983). Although no subjects were alcohol dependent, the scale can be interpreted as providing a continuous measure of harmful alcohol use severity, similar to the AUDIT. IQ was estimated using the National Adult Reading Test (Nelson and Willison, 1991).

### Data analysis

Analyses were conducted using JASP 0.14 software (https://jasp-stats.org/). ANOVA was used to test for group differences with respect to total number of rewards and losses. Effect sizes were calculated using the methods of Cohen's *d* and *r* statistics (Cohen, 1988).

### Neuroimaging data acquisition and preprocessing

Functional whole-brain images were acquired from each participant using a 3T Siemens Tim Trio scanner. Thirty-seven slices were obtained per volume, with an echoplanar imaging sequence comprising a repetition time (TR) 2.5 ms, echo time (TE) 30 ms, flip angle 90°, field of view 22.4 cm, matrix 64 × 64, with a voxel size of 3.5 × 3.5 × 3.5 mm. First, images were visually inspected for artifacts and preprocessed using Statistical Parametric Mapping (SPM; https://www.fil.ion.ucl.ac.uk/spm/). Second, images were realigned and coregistered to the SPM Montreal Neurologic Institute echoplanar template. Finally, the average realigned coregistered image for each subject was used to spatially normalize each realigned coregistered volume and smoothed with an 8 mm full-width half-maximum kernel.

#### Neuroimaging analyses

For a random-effects analysis, data from each subject were analyzed separately (first-level analyses) before summary statistical beta images were tested at the group level (second-level analyses). For first-level analysis, an event-related model-based analysis was implemented with onset regressors at two time points, at the decision time (when the two fractals are presented) and at the outcome delivery time (when the subject saw “you win” or “nothing”). The expected-reward value and the prediction error signals, generated by the optimally fitted SARSA model at the decision and outcome times, respectively, were used to parametrically modulate truncated delta function onset regressors corresponding to the relevant time points, then convolved with the SPM hemodynamic response function, without time or dispersion derivatives. The contrast for analyses extracted only the (RPE and value signal) modulated delta function and not the unmodulated delta functions, which were included in the first-level design matrix to remove the mere effect of these events and not the modulated values that were of interest. As usual we also included realignment parameters as covariates of no interest to covary out any residual head movement not removed by realignment during preprocessing.

For second-level random-effects analyses, summary statistical images from the first-level analyses for each subject were separately entered into second-level analyses to test for within-group activations/deactivations (one group *t* test) and between-group differences (binge drinkers vs controls; two group *t* test). Correlations with binge alcohol use severity (AUDIT and SADQ scales) and mood, anhedonia, and anxiety symptoms [Beck Depression Inventory-II (BDI); State-Trait Anxiety Inventory (STAI)] were also calculated for the binge drinking group alone to test whether symptom severity correlations were consistent with between-group differences. The reason for the correlation analyses was that between-groups differences may be influenced by unrecognized factors, so we sought convergent evidence using binge-drinking-related continuous measures. In addition, correlations with spectroscopy measures (see below) were calculated to test whether variation in these ratios was associated with fMRI activations/deactivations.

Significance was defined as *p* < 0.01 at a whole-brain, familywise-error-corrected level, comprising a simultaneous requirement for a voxel threshold (*p* < 0.05) and a minimum cluster extent (120 voxels) identified using a commonly used Monte-Carlo method. All figures were thresholded at this significance level.

Binge drinkers and controls differed in average age (Table 1). Therefore, we tested whether the between-group differences in ERV and RPE remained significant after controlling for this difference. We repeated the images analyses with age as a covariate. Between-group differences (binge drinkers vs controls) in the brain regions (see below, Results) remained significant with the same significant threshold. Participants' ages did not significantly explain the difference for either ERV or RPE.

**Table 1.** Characteristics of participants

Rating scale and MRS	Controls	Binge drinkers	*p* Values	Friday	Monday	*p* Values
Age	33.7 ± 7.3	22.6 ± 3.5	*p* < 0.001	23 ± 3.3	22.15 ± 3.7	NS
Units of alcohol consumed	1.5 ± 5.7	22.6 ± 8.1	*p* < 0.001	22.3 ± 7.4	22.9 ± 9.0,	NS
Cigarette Smoking	17/19	34/38	ns	17/19	17/19	NS
SADQ	0.4 ± 1.6	8.4 ± 5.3	*p* < 0.001	8.4 ± 5.0	8.3 ± 5.9	NS
AUDIT	0.5 ± 1.7	13.4 ± 4.2	*p* < 0.001	13.2 ± 3.6	13.7 ± 4.9	NS
BDI	2.2 ± 4.4	4.9 ± 0.7	*p* = 0.04	3.9 ± 3.3	5.8 ± 5.6	*p* = 0.03
STAI-S	26.6 ± 8.1	28.4 ± 7.9	NS	27.3 ± 7.7	24.4 ± 8.1	NS
STAI-T	30.7 ± 12.0	34.4 ± 8.8	*p* = 0.03	33.2 ± 9.4	35.5 ± 8.2	NS
GABA/Cr	–	–	–	0.10 ± 0.2	0.08 ± 0.03	*p* = 0.08*^a^*
GLX/Cr	–	–	–	0.06 ± 0.02	0.07 ± 0.01	*p* = 0.04*^a^*
GABA/GLX	–	–	–	1.75 ± 0.85	1.27 ± 0.35	*p* = 0.05*^a^*

AUDIT = Alcohol Use Disorders Identification Test; BDI = Beck Depression Inventory II; Cr = Creatine; GABA = Gamma-amino-butyric acid; GLX = glutamate-glutamine; NS = Non significant; SADQ = Severity of Alcohol Dependence Questionnaire; STAI-S = State-Trait Anxiety Inventory - State; STAI-T = State-Trait Anxiety Inventory - Trait, NS = non significant.

*^a^*Figure 3.

Region of interest (ROI) analyses used the principal eigenvariate as the summary measure of brain response in a 10-mm-diameter sphere.

Mescher-Garwood Point Resolved Spectroscopy (MRS; Mullins et al., 2014) was used to acquire GABA and glutamate-glutamine (GLX) signals, and Gannet software (https://www.gabamrs.com/) was used for analyses. This sequence used TR 1.5 s, TE 68 ms, and ROI 2 × 2.5 × 4 cm^3^ compromising 256 signals for each spectrum. The total spectroscopy acquisition time was 13 min, and the Siemens implementation used chemical shift selective water suppression. The MRS ROI was located in the anterior mid-cingulate cortex, which was chosen as it has been reported to exhibit abnormal functional activity with binge alcohol use and intoxication (Goldstein and Volkow, 2011) and has minimal artifactual signal dropout, unlike more anatomically inferior areas such as the nucleus accumbens.

### Computational modeling of behavior and dopamine function

As with our previous model-based fMRI studies (Gradin et al., 2011, 2014) and studies by independent groups (Pessiglione et al., 2006), we selected the rate (α) and explore/exploit parameter (β) to maximize the log-likelihood of each subject's actual choices according to the model. As with these studies, a single set of parameters was fitted across all groups and subjects as it has been noted (Niv et al., 2006) that multisubject fMRI results are more robust if a single set of parameters is used to generate regressors for all subjects. We used α = 0.45 and β = 3.5 for image analyses as these values were found to be optimal. Briefly, each subject was assumed to be at state $s_{t}$ and selected one of the two fractal stimuli. The task presentation program responded by placing the subject in a new state $s_{t+1}$ and delivering outcome $r_{t+1}$. Subjects aimed to maximize the total number of rewards over time. Here, $Q^{\pi }(s_{t},a)$ is the reward if action *a* is chosen at $s_{t}$ and policy π is followed. The state-action-reward-state-action (SARSA) algorithm improves estimates $\hat{Q}$ of the $Q^{\pi }$ values changing π toward greediness. With SARSA the prediction error depends on the $\hat{Q}$ of the chosen action, and at each time step the SARSA algorithm computes a reward prediction error (RPE) as follows:

$$\delta \left(t+1\right)=r_{t+1}+r\hat{Q}\left(s_{t+1},a^{\prime }\right)-\hat{Q}(s_{t},a),$$

where action *a* is chosen at $s_{t}$, and $a^{\prime }$ is the action chosen at $s_{t+1}$. The prediction error was used to update the estimates of the *Q* values on each trial as follows:

$$\Delta \hat{Q}\left(s_{t},a\right)=\alpha \delta (t+1),$$

where α is the learning rate. Three time points were used in the model, fractal picture presentation time, fractal choice time, and outcome time; and for image analyses two of these time points were used, outcome time δ signal and decision time $\hat{Q}$ value signal of the chosen option. The model calculates the probability of choosing either of the two fractals *x* or *y* on each trial using the softmax rule as follows:

$$p\left(s_{t},a\right)=\frac{e^{\beta \hat{Q}(s_{t},x)}}{e^{\beta \hat{Q}(s_{t},x)}+e^{\beta \hat{Q}(s_{t},y)}},$$

where β is the explore/exploit parameter, and α and β were estimated using a random-effects expectation-maximization method (http://www.quentinhuys.com/tcpw/code/emfit/). For reward-gain trials, the RPE was calculated for the outcome time and the ERV for the decision time with these signals reflecting positive reinforcement.

## Results

### Behavioral analyses

Well-matched behavior between groups is important to ensure comparable engagement with the task and to facilitate interpretation of neuroimaging results. There were no significant differences between binge drinkers and healthy control groups for total number of rewards gained (*p* = 0.2, *d* = −0.3) or total number of losses inadvertently accumulated (*p* = 0.7, *d* = 0.5). There was no significant difference in the number of wins between healthy controls and binge drinkers scanned on Friday and Monday, number of rewards (*p* = 0.1, *d* = 0.6) and number of losses (*p* = 0.9, *d* = −0.2). These differences remained nonsignificant with age as a covariate. The goodness of fit of the behavioral model is defined by the log-likelihood value. The mean log-likelihood fit values were not significantly different (*p* = 0.5, *d* = 0.02) using a two tailed *t* test.

### MRS Spectroscopy

**Figure 3.**
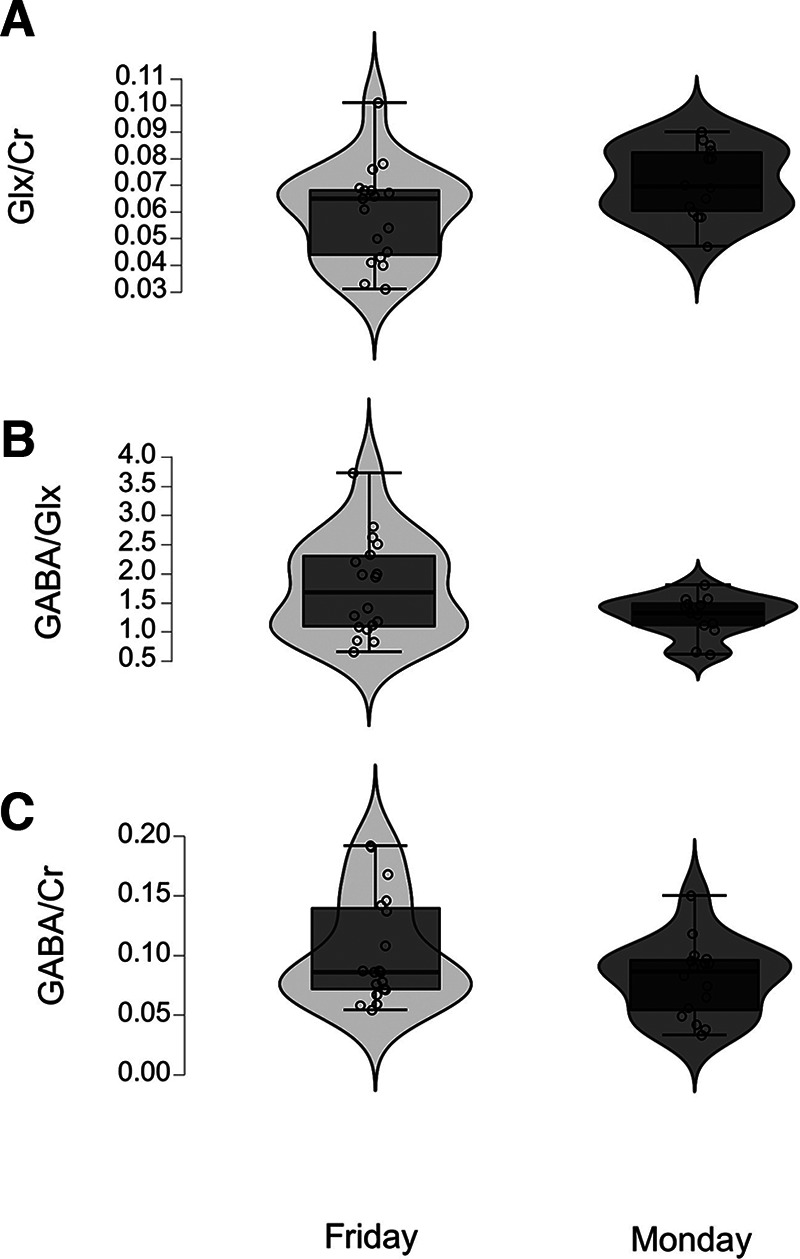
MR spectroscopy results. ***A***, The GLX/Cr differed between binge alcohol drinking groups, with the binge drinkers scanned on Monday having higher ratios (*p* = 0.04, *d* = −0.8). ***B***, The GABA/GLX ratio differed between binge alcohol drinking groups, with the binge drinkers scanned on Friday having slightly higher ratios (*p* = 0.05, *d* = 0.7). ***C***, No significant differences between groups were found for GABA/Cr, but a possible trend (*p* = 0.08, *d* = 0.6) was present.

The GLX/creatine (GLX/Cr) and GABA/GLX ratios differed (*p* = 0.04, *d* = −0.8 and *p* = 0.05, *d* = 0.7, respectively) between binge alcohol drinking groups, with the binge drinkers scanned on Monday having higher and lower ratios respectively (Table 1, Fig. 3). A positive correlation was found between the GLX)/Cr ratio and the number of high value reward choices (*p* = 0.02, *r* = *0.2*). No significant differences between groups were found for GABA/Cr, but a possible trend (*p* = 0.08) was present.

### Expected reward value

**Figure 4.**
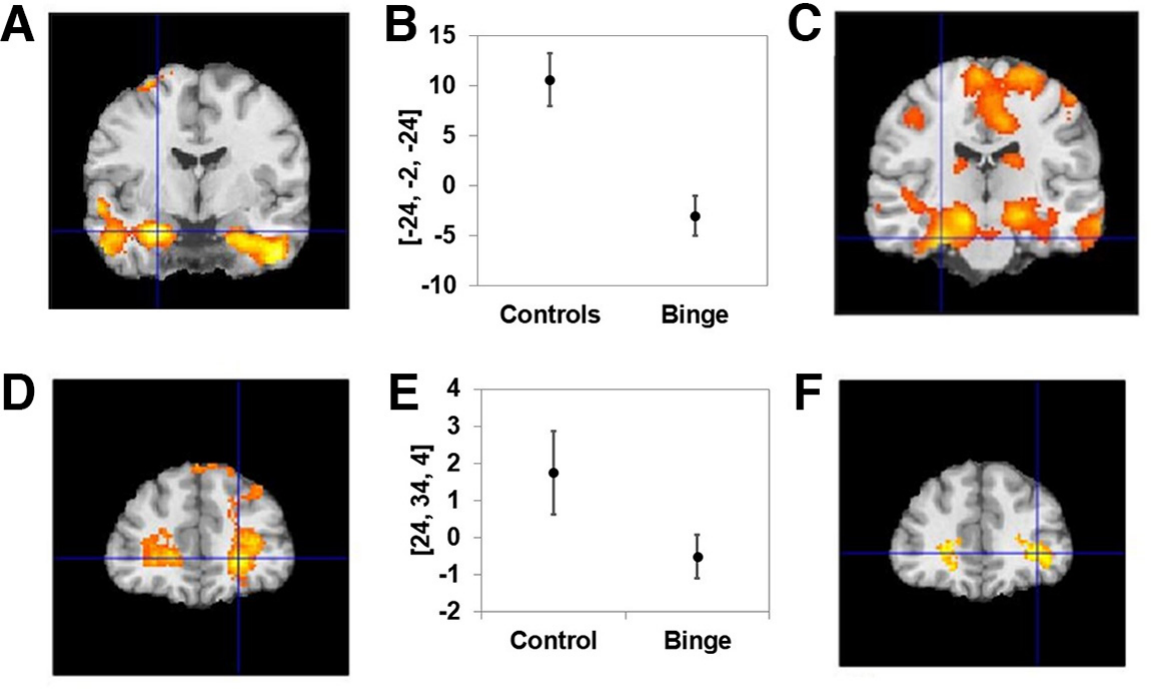
Expected reward value signals. ***A***, Encoding of the ERV signal in the amygdala-hippocampal complex (AHC) in controls with significantly blunted ERV signals in binge drinkers compared with controls. ***B***, Illustration of an ROI centered at the maximally significant AHC voxel. ***C***, AUDIT score significantly negatively correlated with the ERV signal for only the binge drinking group. ***D***, Blunted encoding of the ERV signal in binge drinkers compared with controls in the prefrontal region. ***E***, Illustration with an ROI centered at the maximally significant voxel. ***F***, In binge drinkers alone the GABA/GLX ratio was negatively correlated with the ERV signal. All regions significant at *p* < 0.05, whole-brain corrected.

As predicted the ERV was encoded in the bilateral amygdala-hippocampal complex (−30, −4, −22) *t* = 4.12, (18, −6, −26) *t* = 3.04 and prefrontal region (22, 36, −10) as shown in Figure 4 and Table 2. A two-group *t* test showed ERV encoding was significantly blunted in binge drinkers compared with healthy controls (−36, −12, −24) *t* = 3.08, *d* = 0.9; (24, −6, −26) *t* = 2.35, *d* = 0.8. Additionally, ERV hippocampal encoding for binge drinkers was greater (20, −32, 0) *t* = 3.00 on Friday (with subjects having the longest gap from drinking) compared with Monday (with subjects having the shortest gap from previous drinking).

**Table 2.** Within-group activations and between-group comparisons for expected reward value

Brain region	*x*	*y*	*z*	*t* Value
Controls				
Left amygdala	−30	−4	−22	4.1
Right amygdala	18	−6	−26	3.0
Left orbitofrontal cortex	−22	34	−2	2.9
Right orbitofrontal cortex	26	34	−8	3.0
Controls more than binge drinkers				
Left amygdala	−30	0	−22	4.1
Right amygdala	26	−8	−28	3.2
Left orbitofrontal cortex	−18	36	0	3.4
Right orbitofrontal cortex	22	40	0	3.4

For all binge drinkers there was a negative correlation between ERV amygdala-hippocampal signal strength and (1) AUDIT alcohol scores (−24, 4, −22) *t* = 3.34, (30, −6, −22) *t* = 3.29); (2) State-Trait Anxiety Inventory-State (STAI-S) scores (−28, −14, −22) *t* = 3.2, (28, −22, −22) *t* = 3.5; and (3) the GLX/Cr ratio (−36, 4, −34) *t* = 3.43 correlated with the prefrontal ERV; and (4) the GABA/GLX ratio (34, 40, 0) *t* = 3 and prefrontal ERV signals significantly correlated (Fig. 4). GLX/Cr and GABA/GLX ratios were reduced in binge drinkers in general. For binge drinkers scanned on a Monday, ratings of problematic alcohol use (SADQ and AUDIT) negatively correlated with ERV signals in the amygdala-hippocampal complex (−14, −16, −18) *t* = 3.9, (18, −8, −28) *t* = 3.4.

In summary, ERV encoding was blunted in the amygdala-hippocampal complex of binge drinkers compared with controls, and increased binge drinking ratings and spectroscopic abnormalities were associated with increased blunting of ERV encoding within binge drinkers alone.

### Reward prediction error signals

**Figure 5.**
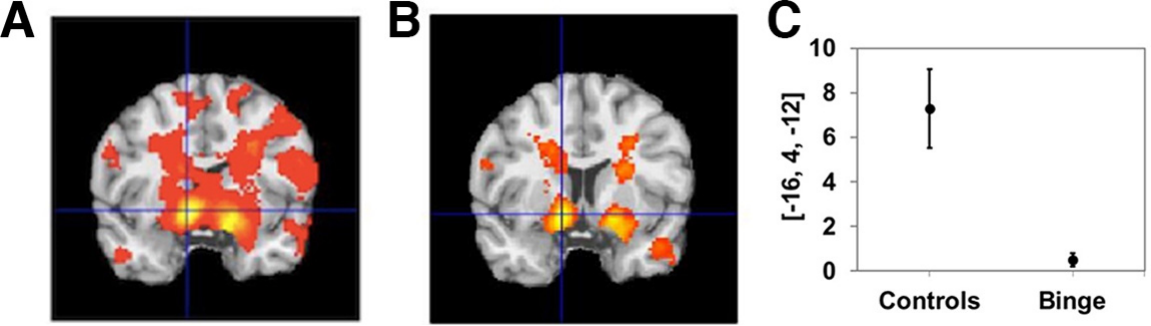
Reward prediction error signals. ***A–C***, RPE signal encoding in the accumbens of controls (***A***), with (***B***) significantly blunted RPE encoding in binge drinkers compared with controls, also shown (***C***) as an ROI centered at the maximally significant voxel. All regions significant at *p* < 0.05, whole-brain corrected.

As expected, RPE signals were encoded in the nucleus accumbens of controls (−12, 8, −6) *t* = 7.8, (12, 8, −14) *t* = 6.81; subgenual cingulate cortex (2, 50, −14) *t* = 5.03; and posterior cingulate (4, −45, 30) *t* = 4.21 (Fig. 5, Table 3). Similarly for binge drinkers, RPE signals were present in the bilateral accumbens (−10, 12, −8) *t* = 2.5, (10, 12, −8) *t* = 2.5; subgenual cingulate cortex (6, 50, −18) *t* = 4.8; and posterior cingulate (6, −34, 40) *t* = 3.3 (Table 3). Compared with controls, binge drinkers exhibited significantly blunted RPE signals in the nucleus accumbens (−14, 8, −8) *t* = 5.46, d = 0.8; (14, 8, −12) *t* = 4.96; and posterior cingulate (0, −46, 26) *t* = 3.05 (Fig. 5, Table 3). For all binge drinkers (combined Friday and Monday groups), the AUDIT score negatively correlated with RPE nucleus accumbens (−16, 14, −10) *t* = 3.6, (10, 18, −12) *t* = 3.6 signal strength and STAI-S (−22, 8, −3) ratings.

**Table 3.** Within-group activations and between-group comparisons for reward prediction error signals

Brain region	*x*	*y*	*z*	*t* Value
Controls				
Left nucleus accumbens	−12	8	−6	7.80
Right nucleus accumbens	12	8	−14	6.81
Right subgenual cingulate cortex	2	50	−14	5.03
Right posterior cingulate cortex	4	−45	30	4.21
Binge Drinkers				
Left nucleus accumbens	−10	12	−8	2.5
Right nucleus accumbens	10	12	−8	2.5
Right subgenual cingulate cortex	6	50	−18	4.8
Right posterior cingulate cortex	6	−34	40	3.3
Controls more than binge drinkers				
Left nucleus accumbens	−14	8	−8	5.46
Right nucleus accumbens	14	8	−12	4.96
Posterior cingulate cortex	0	−46	26	3.05

## Discussion

Addiction has been formulated as an aberrant type of associative learning (McClure et al., 2003; Redish, 2004). A common feature of different types of associative learning is that dopamine firing at the time of the reward [unconditioned stimulus (US)] diminishes, and dopamine firing at the time of the cue [conditioned stimulus (CS)] predicting US delivery increases (Fig. 1; Kumar et al., 2008; Gradin et al., 2011). There is robust experimental evidence in healthy animals and humans for cue-induced dopamine release for natural reinforcers (Schultz et al., 1997; Contreras-Vidal and Schultz, 1999; Pessiglione et al., 2006). Associating learning is quite specific to the cues and reinforcers used during learning (Schultz et al., 1997; Contreras-Vidal and Schultz, 1999; Niv et al., 2005; Pessiglione et al., 2006; Chase et al., 2015).

Redish (2004) proposed that drug associative learning can be modeled using a TD approach with pharmacological enhancement of dopamine release at the time of drug delivery, causing enhancement of the ERV of the drug. McClure et al. (2003) identified the psychological concept of incentive salience (Robinson and Berridge, 1993) with the computational notion of ERV, suggesting that TD theory formalizes incentive-sensitization ideas about attributing incentive salience through a boosting process. However, Robinson and Berridge (1993) favor a more complex view of incentive salience, proposing the ERV is transformed to a different motivational value, with ERV and motivation potentially dissociable (Berridge, 2012). Experimentally, as noted earlier, for humans with alcohol or other drug dependency, drug cue exposure is associated with dopamine release (Volkow et al., 2006; Wong et al., 2006; Cox et al., 2017), which has been linked to subjective craving (Sell et al., 2000; Volkow et al., 2006; Saunders et al., 2013). Dopamine release at the time of drug delivery is in contrast blunted (Volkow et al., 1997; Martinez et al., 2005, 2007). These observations appear consistent with TD theory (Fig. 1). Furthermore, preclinical work suggests that a small dopamine peak (ERV) on a blunted tonic dopamine background (because of allostatic reward blunting) is much more salient than on a normal tonic dopamine background (Koob and Le Moal, 1997). Notably, as proposed by Keiflin and Janak (2015), the concept of persistent dopamine-RPE is a key hypothesis for drug addiction.

In addition, alcohol and drug dependency are associated with increased time spent obtaining and using alcohol/drugs but also self-neglect. This suggests that although alcohol/drug cues are associated with increased salience and dopamine activity (Volkow et al., 2006; Wong et al., 2006; Cox et al., 2017), non-drug/alcohol-related stimuli become undervalued (Koob and Volkow, 2010; Zilverstand et al., 2018), implying decreased motivational value of natural rewards. In addition, alcohol/drug dependency is associated with reduced attention to natural rewards (Volkow et al., 2004; Koob and Volkow, 2010). Here, we tested the hypothesis that ERV signals, for non-alcohol-associated cues, were blunted in binge alcohol drinkers. The RPE signal is defined as *r* − ERV (Fig. 1), and previous analyses of our fMRI data using *r* showed blunting of this signal in binge drinkers (Tolomeo et al., 2021). From the perspective of TD theory, this implies the RPE signal should also be blunted and consequently the ERV signal at the nonalcohol cue time. Our experimental results support this hypothesis.

Regarding our second hypothesis of syndrome severity measures correlating with brain activity consistent with between-groups findings, increased severity of binge drinking quantified by AUDIT scores and higher ratings of anxiety were associated with increased blunting of the ERV in the amygdala/hippocampus. The hippocampus has been linked to craving and alcohol preoccupation, and the extended amygdala, comprising the central nucleus of amygdala, bed nucleus of stria terminalis, and accumbens shell is important for adverse effects on reward function produced by stress driven by compulsive alcohol use (Koob and Le Moal, 2008). Increased GLX/Cr ratios and GABA/GLX ratios were associated with increased blunting of the ERV in the prefrontal cortex, supporting our third hypothesis. The results of our present analyses imply that the motivational importance (reflected by ERV) of nonalcohol rewards is blunted in binge drinkers. We conceptualized binge drinking as on a continuum with alcohol dependency (Fig. 1), so the prediction of this effect should be more pronounced in alcohol-dependent individuals. Additionally, choosing between options that differ in terms of expected reward values may occur in the brain by a mutual inhibition competition mechanism, a hypothesis tested in healthy subjects using computational modeling of learning behavior, fMRI, and GABA and glutamate spectroscopy (Jocham et al., 2012). Consistent with this, the authors reported that a model parameter, the softmax inverse temperature, correlated with GABA and glutamate concentrations (Jocham et al., 2012). There is robust preclinical evidence for altered concentration of these neurotransmitters in alcohol-dependent animals, and in the present study, we found consistent evidence for spectroscopic abnormalities in binge drinking humans. This implies that abnormal GABA and glutamate concentrations could be directly linked to abnormal non-alcohol-related value encoding observed in binge drinking and alcohol-dependent humans. More work is required to address this hypothesis.

Clinically, abstinence is relatively easy to achieve; however, achieving sustained abstinence is extremely difficult and arguably represents the biggest problem for advancing addiction medicine. The two commonest causes of relapse are stress-induced relapse and alcohol/drug-cue-induced relapse, with the former being by far the most common cause (Marlatt, 1978). In our view, allostasis theory and TD theory applied to addiction explain different and complementary features of addiction. Allostasis theory describes how aversive experiences associated with negative valence system activation are enhanced in addiction (Tolomeo et al., 2021), emphasizing the crucial importance of negative reinforcement in sustaining addiction and causing enduring vulnerability to relapse once abstinence has been achieved (Koob, 2009). Allostasis theory provides, in our view, the best framework for studying stress-induced relapse and discovery of new effective treatments addressing this problem. However, allostasis theory is not good at explaining the less common cue-induced relapse, which has been hypothesized to be caused by alcohol/drug-predicting cues having (chemically enhanced) value encoded in the dopamine system because of repeated alcohol/drug reward learning (Redish, 2004). In our view these theories may be reconciled by hypothesizing that for nonalcohol/drug rewards, the binary reward response (*r*) is blunted (because of allostasis), leading to (by TD theory) blunted RPE and blunted cue valuation signals. The results of our present study are consistent with this hypothesis. In contrast, for the case of alcohol/drug rewards, we hypothesize that the direct chemical effect on dopamine and other systems results in enhanced alcohol/drug cue valuation (Redish, 2004), overriding allostatic reward blunting. Supporting this is evidence from PET studies on patients with addiction reporting enhanced striatal signals at the time of cue exposure and blunted signals at the time of alcohol/drug delivery (Volkow et al., 1997, 2006). Blunted striatal responses to the delivery of nonalcohol/drug rewards are predicted by both TD learning and allostasis theories in addiction.

The strengths of our present study include the use of computational modeling to test for functional brain abnormalities in binge drinkers without confounding brain structure abnormalities that would be present in alcohol-dependent individuals. One limitation is that it was not practical to also test alcohol cue responses in the same subjects as it was beyond the scope of the present study. However, we predict these would be increased, consistent with PET studies. Additionally, the ERV might in some situations be dissociable from subjective motivation; however, our study was not designed to test this theory. The present work has focused on fMRI signals consistent with cue-induced dopamine release because of its link to craving and relapse. However, two-thirds of relapse to alcohol use disorder is because of stress (Marlatt, 1978), namely, hyperkatefia, with many other neurotransmitters and systems implicated (Koob and Schulkin, 2018). Another potential limitation is that the average age of binge drinkers was significantly less than for controls; therefore, we tested whether between-group differences for ERV and RPE remained significant after controlling for age.

In summary, using task-based event-related fMRI, previously we tested hypotheses derived from allostasis theory reporting results consistent with predictions (Tolomeo et al., 2021). Here, we analyzed these same data using a TD-model-based fMRI approach and reported blunted non-alcohol-related ERV cue signals in binge alcohol drinkers. A better understanding of the mechanisms of harmful alcohol use will facilitate the development of better treatments, which should aim to decrease the motivational value of alcohol and increase the motivational value of non-alcohol-related stimuli.
